# Perfusion and Structural Impairment in Minor Stroke and Transient Ischemic Attack With Intracranial Atherosclerotic Stenosis: Associations With Cognitive Decline

**DOI:** 10.1002/cns.70693

**Published:** 2025-12-09

**Authors:** Meiling Shang, Gezhi Yan, Wanghuan Dun, Fude Liu, Ling Ma, Lu Quan, Fernando Pico, Shiliang Jiang, Xiaotong Chi, Bingbing Guo, Huiping Liu, Zepeng Tian, Peizhang Yan, Xuan Niu, Jingmei Xie, Jianfeng Han, Ming Zhang

**Affiliations:** ^1^ Department of Medical Imaging The First Affiliated Hospital of Xi'an Jiaotong University Xi'an Shaanxi Province China; ^2^ School of Future Technology Xi'an Jiaotong University Xi'an Shaanxi Province China; ^3^ Deparment of Neurology The First Affiliated Hospital of Xi'an Jiaotong University Xi'an Shaanxi Province China; ^4^ Department of Rehabilitation Medicine The First Affiliated Hospital of Xi'an Jiaotong University Xi'an Shaanxi Province China; ^5^ Versailles Saint‐Quentin‐en‐Yvelines and Paris Saclay University Paris France; ^6^ Zonglian College Xi'an Jiaotong University Xi'an Shaanxi Province China; ^7^ The First Affiliated Hospital of Xi'an Jiaotong University, Yulin Hospital Yulin Shaanxi Province China

**Keywords:** cognition, intracranial atherosclerotic stenosis, minor stroke, perfusion, structural impairment, transient ischemic attack

## Abstract

**Aims:**

To investigate the characteristics of hypoperfusion and structural impairment in patients with transient ischemic attack (TIA) or minor stroke (MS) with intracranial atherosclerotic stenosis (ICAS) and evaluate its impact on cognitive decline.

**Methods:**

Cognitive function in 47 patients and 33 health controls (HC) was assessed using the Montreal Cognitive Assessment (MoCA) tool. Arterial spin labeling with two distinct postlabeling delays and 3D T1 imaging was conducted to assess cerebral blood flow (CBF), morphometric features, and asymmetry index (AI).

**Results:**

Compared with HC, both the left and right involved patients showed reduced scores for total MoCA score. Comparisons of CBF with HC revealed that significant ischemic areas in patients were primarily localized to the perfusion territory of the middle cerebral artery in the affected hemisphere (*p* < 0.05, family‐wise error [FWE] corrected). Despite morphometry abnormality being locally confined, AI exhibits more widespread alterations across a wider range of brain regions (both *p* < 0.05, FWE corrected). Both hypoperfusion and structural impairment were significantly associated with reduced MoCA score in left‐involved patients, whereas only hypoperfusion showed a significant association in those with right‐hemisphere involvement (*p* < 0.05, FWE corrected). Furthermore, the effect of ischemia on cognition was mediated by structural impairment and compensatory CBF simultaneously.

**Conclusion:**

These findings highlight that hypoperfusion and structural impairment are already concerning and jointly associated with cognitive impairment in MS/TIA patients with ICAS, emphasizing the need for early detection and intervention.

## Graphical abstract

Despite owning well‐developed compensatory circulation, the perfusion, structural integrity, and cognition impairment of minor stroke/transient ischemic attack patients combined with intracranial atherosclerotic stenosis are already in an alarmed condition to get more attention from clinical therapy.

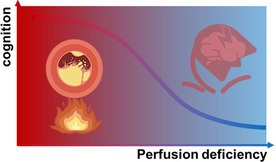

## 1 Introduction

Minor strokes (MS) and transient ischemic attacks (TIA) often precede major strokes, greatly elevating the risk of future cerebrovascular events, particularly in patients with severe intracranial atherosclerotic stenosis (ICAS), where the risk doubles [1, 2]. MS/TIA patients with ICAS require intensive secondary stroke prevention, given their poorer collateral status compared with subjects with asymptomatic ICAS [2, 3, 4]. Nevertheless, the pathophysiological characteristics in these MS/TIA patients with ICAS remain poorly understood.

The presence of large artery atherosclerotic stenosis is the culprit lesion for the ischemic symptoms in these patients. Fortunately, the chronic progression of stenosis often facilitates the development of a broader collateral circulation, which can potentially mitigate the severity of functional impairment [5, 6]. This phenomenon is relevant in this particular population, as the initial event does not result in substantial structural damage or functional loss [7]. However, the relationship between compensatory perfusion and clinical symptoms remains unclear and requires further clarification.

Patients with MS/TIA or ICAS may already exhibit early cognitive impairments across multiple domains [8, 9]. Studies have shown that hypoperfusion is an independent risk factor for vascular cognitive impairment, which can trigger mechanisms of cognitive decline, such as hypoxia, inflammatory responses, and even microinfarctions in cortical and subcortical regions [10, 11]. However, research on the extent of cognitive impairment and its relationship with hypoperfusion in MS/TIA patients with ICAS is lacking. Additionally, the protective role of compensatory perfusion, potentially supported by well‐developed collateral circulation, remains unvalidated.

Cortical morphological changes may occur early in the course of reduced perfusion, disrupting the structural foundation necessary for normal cognitive function [12]. Although a well‐developed collateral circulation might alleviate the severity of ischemia and structural impairment, the extent of gray matter damage and its relationship to cognitive decline require further investigation in these patients [13]. Varying patterns of cortical atrophy and compensatory phenomena can also alter gray matter asymmetry, which may provide new insights regarding cognitive impairment in this population [14, 15, 16]. While studies have preliminarily explored the impact of perfusion or structural damage on cognition, few have considered the role of structural integrity in modulating the relationship between perfusion and cognitive function within these MS/TIA patients with ICAS [10, 11, 12]. In this context, multimodal neuroimaging could offer crucial insights into the central mechanisms of cognitive impairment.

To accurately characterize the disease features and brain health status of MS/TIA patients with ICAS, we utilized arterial spin labeling (ASL) with two distinct postlabeling delays and high‐resolution structural magnetic resonance imaging (MRI) to examine both perfusion and gray matter structure in this cohort at high risk for stroke. We also employed the Montreal Cognitive Assessment (MoCA) screening tool to evaluate overall cognitive function, aiming to establish the interrelationships between ischemia, compensatory perfusion, cortical structure, and cognitive performance.

## 2 Methods

### 2.1 Participants

This study was approved by the Institutional Review Board of the First Affiliated Hospital of the Medical College in Xi'an Jiaotong University (XJTU1AF2022LSL‐023), following the requirements of the Helsinki Declaration. Informed consent was completely understood and signed by all participants. Patients were recruited from the neurology department of the First Affiliated Hospital of Xi'an Jiaotong University and health controls (HC) were recruited openly at the meantime. The inclusion criteria of patients were: (1) diagnosis of acute MS or TIA for the first time; (2) confirmed severe stenosis or occlusion (70%–100%) of unilateral large intracranial arteries in the anterior circulation and none to mild stenosis (0%–50%) of contralateral arteries by digital subtraction angiography (DSA) within 2 weeks; (3) evaluated using National Institute of Health stroke scale (NIHSS) with the total score ≤ 3; (4) undergone only pharmacotherapy and no endovascular treatment; (5) aged 18–80 years; (6) and right—handedness. Patients would be excluded for the following reasons: (1) progressive stroke; (2) history of severe systemic or neuropsychiatric disease; or (3) any contraindications for MRI scans. According to the Chinese ischemic stroke subclassification [17], ICAS was identified as the primary cause of their ischemic cerebrovascular events with the evidence of vessel involvement, clinical evaluation of neurological signs and exclusion of other neurological diseases. After recruitment, these patients were divided into two groups based on the hemisphere with ICAS (patients with left/right hemisphere involvement = 29/18). HC were recruited as per the following criteria: (1) aged 18–80 years; (2) right—handedness; (3) no organic or neuropsychiatric disease history; (4) MoCA score ≥ 23 (the criteria were adopted based on the latest validated study to improve the accuracy in detecting mild cognitive impairment [18], differing from the commonly recommended cutoff values of 10, 18, 26 for detecting severe, medium or mild cognitive impairment, respectively); and (5) no contraindications for MRI scans.

### 2.2 Neurological Assessments

The NIHSS was used to evaluate the neurological impairment of participants. Since the diagnostic criteria for MS are not standardized, the commonly used cutoff value of 3 was employed in this study, based on previous studies [19]. Developed by Nasreddine in 2005 [20], MoCA is a widely accepted screening tool for evaluating cognitive impairment and was used in this study to assess the cognitive performance of both patients and HC. Subitem scores were calculated separately for analysis. The neurological assessments of all participants were performed by a neurologist on the same day of MRI acquisition.

### 2.3 MRI Data Acquisition

For patients, scanning procedures were scheduled no later than 2 weeks after the first onset of MS/TIA. Three‐dimensional T1 brain volume (3D T1 BRAVO) and pseudo‐continuous arterial spin labeling (3D pCASL) sequences were utilized. Scanning parameters are listed in the Supporting Information.

### 2.4 MRI Data Preprocessing

#### 2.4.1 Cerebral Blood Flow Measurement

All 3D pCASL image postprocessing was performed using the application FuncTool in Advanced Workstation AW4.6 (GE HealthCare, Chicago, IL, USA) to subtract out cerebral blood flow (CBF) maps. The CBF maps were registered and normalized to a Montreal Neurological Institute template using statistical parametric mapping software (SPM12, University College of London, available at www.fil.ion.ucl.ac.uk/spm/software/Spm12). The normalized images were then smoothed with an isotropic Gaussian kernel of 8 × 8 × 8 mm^3^ full‐width at half maximum (FWHM). Compensatory perfusion conditions were assessed by subtracting CBF maps at PLD = 1.5 s from those at PLD = 2.5 s [21].

#### 2.4.2 Cortical Morphology Measurement

Gray matter volume (GMV) and thickness were measured using FreeSurfer V7.3.2 online toolkit (http://surfer.nmr.mgh.harvard.edu/, see Supporting Information), utilizing the standard recon‐all pipeline for cortical reconstruction and volumetric segmentation.

#### 2.4.3 Gray Matter Asymmetry Measurement

Gray matter asymmetry was presented as an asymmetry index (AI) in accordance with a well‐recognized calculation paradigm [22]. Specifically, the structural images of all participants were flipped at first. All original and flipped images were warped and normalized to a symmetrical DARTEL template, which was generated based on the current dataset. AI was calculated based on the formula below:

$$\mathrm{AI}=\frac{2\left(i1-i2\right)}{i1+i2}*i3$$

where *i*1 refers to the warped original image, *i*2 refers to the warped flipped image, and *i*3 refers to a left hemisphere mask. By retaining left hemisphere values while setting right hemisphere values to zero, the incorporation of *i*3 is intended to eliminate the mirrored (i.e., sign‐reversed) AI values of the right hemisphere. This ensures that only nonredundant AI values are retained and mapped onto the left hemisphere, making the resulting AI matrix more appropriate for subsequent multiple comparison corrections. After calculation, the left hemisphere was conserved, in which AI > 0 indicated leftward asymmetry, while AI < 0 indicated rightward asymmetry. Smoothing of the AI images was performed using an isotropic 8 mm × 8 mm × 8 mm Gaussian kernel FWHM.

### 2.5 Statistical Analysis

SPSS version 20, SPM12, and FreeSurfer were used for the comparison and correlation analyses. The mediation and moderation analyses were conducted using the PROCESS version 2.16 tool. The statistical methodology is discussed in detail in the Supporting Information.

## 3 Results

### 3.1 Demographics and Clinical Data

In total, 47 patients and 33 HCs participated in the current study. The patient cohort included 28 patients with left hemisphere involvement and 19 patients with right hemisphere involvement. Left/right involved patients differed statistically in the NIHSS score (*p* = 0.01), distribution of offending artery (*p* = 0.04), and qualifying event (*p* = 0.007), whereas other demographic and clinical characteristics did not differ significantly (Table 1).

**TABLE 1:** Demographic and clinical characteristics of patients and HC.

	Patients (*n* = 47)		HC (*n* = 33)	*p*
	L (*n* = 28)	R (*n* = 19)		
Age	49.7 ± 11.5	50.2 ± 12.9	51.5 ± 9.3	0.79
Female	11 (39.3%)	4 (21.1%)	16 (48.5%)	0.15
Education	9 (9, 12)	12 (9, 12)	12 (10–14)	0.05
**Cerebrovascular risk factors**				
BMI	24.8 ± 2.8	25.4 ± 2.7	24.3 ± 2.5	0.41
Smoke	11 (39.3%)	9 (47.4%)	8 (24.2%)	0.20
Alcohol	7 (25.0%)	5 (26.3%)	5 (15.1%)	0.53
Diabetes	7 (25.0%)	3 (15.8%)	—	0.45
Hypertension	20 (71.4%)	12 (63.2%)	—	0.55
Hyperlipidemia	9 (32.1%)	6 (31.6%)	—	0.97
HbA1c (%)	5.8 (5.5–6.9)	6 (5.6–6.6)	—	0.73
HCY (μmol/L)	16.3 (14–24.7)	13.3 (10.2–21.1)	—	0.07
**NHISS score**	0 (0–2)	0 (0–0)	—	0.01*
**Offending vessel**				
ICA	17 (60.7%)	5 (26.3%)	—	0.04*
MCA	11 (39.3%)	14 (73.7%)	—	
**Stenosis, ipsilateral**				
Severe (70%–99%)	10 (35.7%)	9 (47.4%)	—	0.55
Occlusion	18 (64.3%)	10 (52.6%)	—	
**Stenosis, contralateral**				
None	17 (60.7%)	13 (68.4%)	—	0.76
Minor (0%–49%)	11 (39.3%)	6 (31.6%)	—	
**Qualifying event**				
TIA	8 (28.6%)	13 (66.7%)	—	0.007**
Minor stroke	20 (71.4%)	6 (33.3%)	—	

*Note:* Data are presented as mean ± standard deviation for normally distributed data, median (interquartile range) for abnormally distributed data, or *n* (%) for count data.

Abbreviations: BMI, body mass index; HbA1c, hemoglobinA1c; HC, health controls; HCY, homocysteine; ICA, internal carotid artery; L, left hemisphere involvement; MCA, middle cerebral artery; NHISS, National Institutes of Health Stroke Scale; R, right hemisphere involvement; TIA, transient ischemic attack.

*p < 0.05; **p < 0.01.

As shown in Table 2, comparison among the three groups revealed significant differences in the total MoCA score and most subitems (*p* < 0.05), except for “Naming.” In post hoc analyses, patients with left hemisphere involvement exhibited significantly lower scores than HC across all variables that showed significant differences in the overall three‐group comparison (*p* < 0.0167, Bonferroni corrected). In contrast, patients with right hemisphere involvement showed significantly lower scores than HC in the subitems “Language,” “Abstraction,” and “Orientation” (*p* < 0.0167, Bonferroni corrected). In addition, patients with left hemisphere involvement scored significantly lower than those with right hemisphere involvement on the “Attention” subitem (*p* < 0.0167, Bonferroni corrected).

**TABLE 2:** Comparisons of total MoCA score and subitems [median (interquartile range)].

	L	R	HC	*p* *
Total MoCA score	22 (12, 24)^a^	25 (22, 27)^a^	28 (25, 30)	< 0.001
Visuospatial/executive function	3 (2, 5)^a^	4 (3, 5)	5 (5, 5)	< 0.001
Naming	3 (2, 3)	3 (2, 3)	3 (3, 3)	0.096
Attention	5 (2, 6)^a^, ^b^	6 (6, 6)	6 (6, 6)	< 0.001
Language	1 (1, 2)^a^	2 (1, 2)^a^	3 (2, 3)	< 0.001
Abstraction	1 (0, 2)^a^	1 (0, 2)^a^	2 (2, 2)	< 0.001
Delayed recall	2 (0, 3)^a^	3 (0, 4)	3 (2, 4)	0.047
Orientation	5 (4, 6)^a^	6 (5, 6)^a^	6 (6, 6)	< 0.001

Abbreviations: HC, health control; L, patients with left hemisphere involvement; MoCA, Montreal Cognitive Assessment; R, patients with right hemisphere involvement.

^a^ *p* < 0.0167, compared with healthy controls.

^b^ *p* < 0.0167, compared with patients with right hemisphere involvement.

* *p* value for the comparison among the three groups.

### 3.2 CBF Abnormalities

**FIGURE 1:**
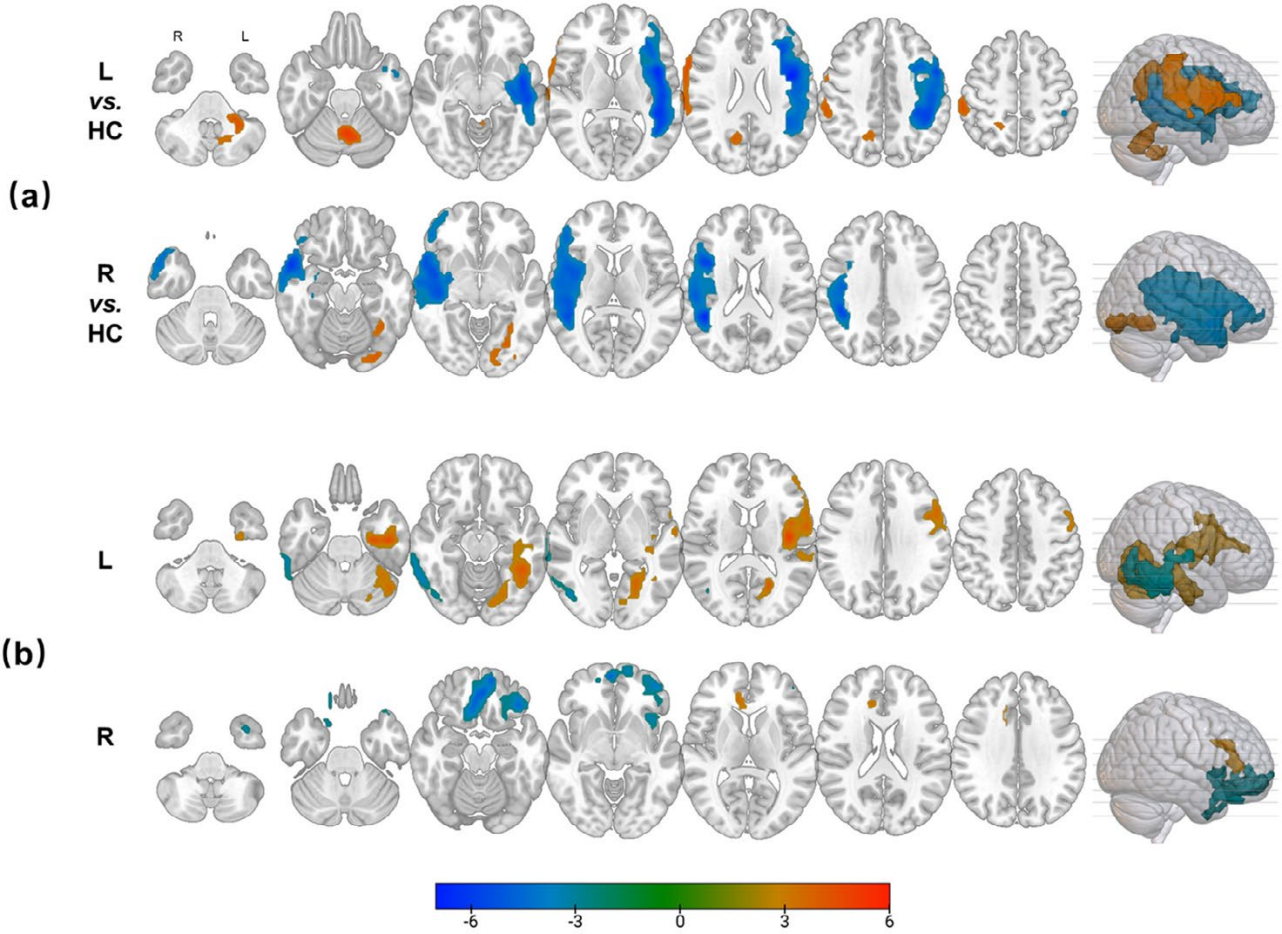
Differentiated brain regions with abnormal cerebral blood flow (CBF) and correlation with total MoCA score. (a) Hypoperfused and hyperfused clusters. (b) Correlation between CBF and total MoCA score. HC, healthy controls; L, patients with left hemisphere involvement; R, patients with right hemisphere involvement.

Compared with HC, patients exhibited significantly decreased CBF in a wide region encompassing the temporal, dorsolateral prefrontal, motor, and inferior parietal cortices in the affected hemisphere (cluster‐level *p* < 0.05, family‐wise error [FWE] corrected). This decrease roughly corresponded to the perfusion map of the middle cerebral artery. Additionally, perfusion was increased in the left cerebellum and regions approximately symmetrical to the ischemic areas in the right hemisphere in patients with left hemisphere involvement (cluster‐level *p* < 0.05, FWE corrected), as well as in the left fusiform and occipital cortices in patients with right hemisphere involvement (cluster‐level *p* < 0.05, FWE corrected) (Figure 1a).

Correlation analysis revealed a significant positive correlation between the total MoCA score and the CBF in the affected dorsolateral prefrontal cortex, motor cortex, occipital cortex, and cerebellar cortex of patients with left hemisphere involvement (cluster‐level *p* < 0.05, FWE corrected). Additionally, a borderline statistically significant negative correlation was found between the CBF and total MoCA score in the temporal, occipital, and cerebellar regions of the right hemisphere (cluster‐level *p* = 0.06, FWE corrected). For patients with right hemisphere involvement, a significant positive correlation was found between the total MoCA score and the CBF in the right anterior cingulate cortex (cluster‐level *p* < 0.05, FWE corrected). Conversely, the total MoCA score was significantly negatively correlated with the CBF in the left frontal and right rectus cortices (cluster‐level *p* < 0.05, FWE corrected) (Figure 1b).

### 3.3 Gray Matter Abnormality

Compared with HC, a larger GMV was found in the right superior temporal cortex in patients with left hemisphere involvement (cluster‐level *p* < 0.05, FWE corrected, Figure S1a), whereas those with right hemisphere involvement showed increased GMV in the right postcentral cortex (cluster‐level *p* < 0.05, FWE corrected, Figure S1b). For patients with left hemisphere involvement, the total MoCA score was positively correlated with cortical thickness in the left superior temporal cortex and banks of the superior temporal sulcus and negatively correlated with the cortical thickness of the left pericalcarine cortex and right precuneus cortex (cluster‐level *p* < 0.05, FWE corrected, Figure S1c). Additionally, the total MoCA score was negatively correlated with the GMV of the left superior and middle frontal cortices, left precuneus as well as right superior occipital cortex (cluster‐level *p* < 0.05, FWE corrected, Figure S1d). However, for patients with right hemisphere involvement, no significant correlation was found between total MoCA score and either GMV or cortical thickness.

### 3.4 Decreased Asymmetry Trend

**FIGURE 2:**
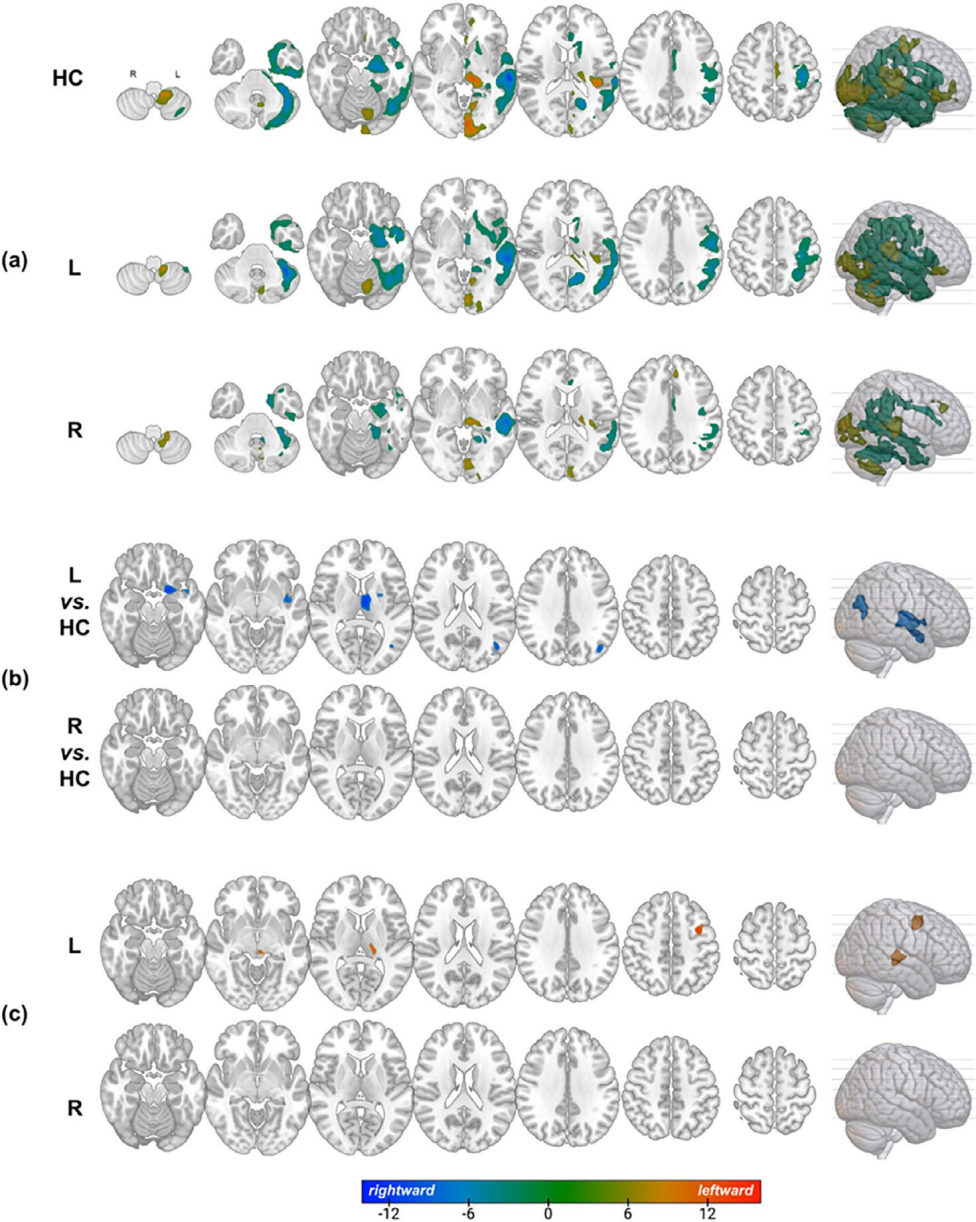
The distribution and comparison results of AI and the correlation between AI and total MoCA score. (a) AI distribution in patients with left or right hemisphere involvement and healthy controls. (b) Comparison between patients with left/right hemisphere involvement and healthy controls. (c) Correlation between AI and the total MoCA score. HC, healthy controls; L, patients with left hemisphere involvement; R, patients with right hemisphere involvement.

Single‐sample *t*‐tests were used to illustrate asymmetry trends in the two patient groups and HC, respectively. The results are presented in Figure 2a and described in the Supporting Information.

A two‐sample *t*‐test indicated that, compared with HC, in patients with left hemisphere involvement, the AI value is decreased in the superior temporal pole, parahippocampal cortex, amygdala, insula, putamen, and thalamus (cluster‐level *p* < 0.05, FWE corrected). An increased AI in patients with right hemisphere involvement suggests a similar trend (cluster‐level *p* < 0.05, FWE corrected). However, no significant decrease or increase in AI was found when comparing patients with right hemisphere involvement to HC, although the scope of AI was significantly reduced in the one‐sample *t*‐test results (Figure 2b).

A significant positive correlation was found between total MoCA score and AI in the affected thalamus and middle frontal and precentral cortex in patients with left hemisphere involvement (cluster‐level *p* < 0.05, FWE corrected). No significant differences were observed in patients with right hemisphere involvement (Figure 2c).

### 3.5 Correlation Between Hypoperfusion, Compensatory CBF, and Gray Matter Atrophy

**FIGURE 3:**
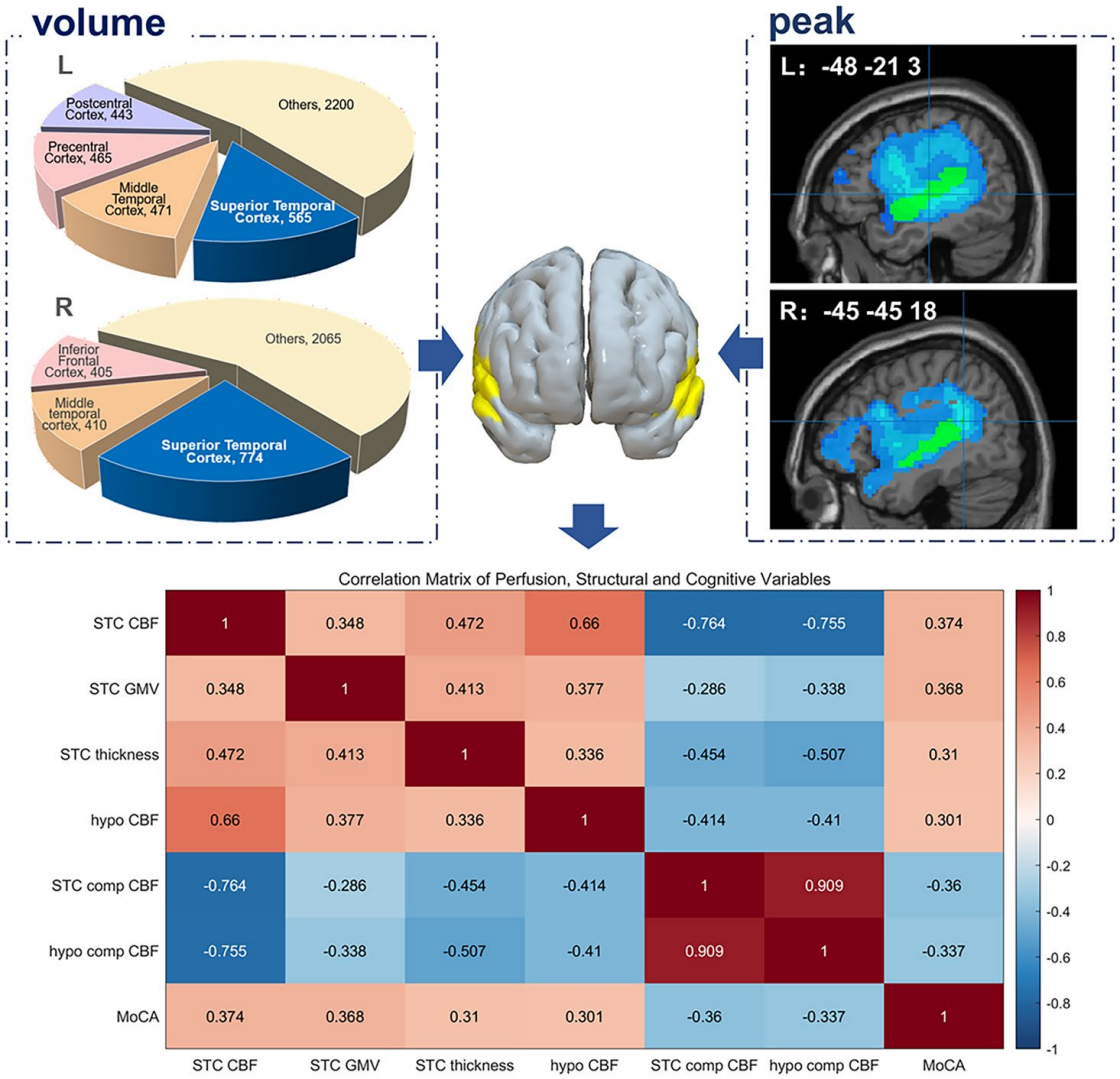
Volume structure (in voxels), peak location of ischemic region, and correlation matrix of perfusion, structural, and cognitive variables. The numbers in the correlation matrix represent *r* values for each pairwise analysis. hypo CBF, cerebral blood flow in hypoperfused area; hypo comp CBF, compensatory cerebral blood flow in hypoperfused area; L, patients with left hemisphere involvement; MoCA, Montreal Cognitive Assessment; R, patients with right hemisphere involvement; STC CBF, cerebral blood flow in involved superior temporal cortex; STC comp CBF, compensatory cerebral blood flow in superior temporal cortex; STC GMV, gray matter volume of involved superior temporal cortex; STC, superior temporal cortex.

Based on the results of previous comparisons, the affected superior temporal cortex represented the ischemic core with the peak location and the maximum number of affected voxels. This result is consistent in patients with left and right hemisphere involvement (Figure 3). Based on these findings, we further investigated the correlation between gray matter atrophy in the affected superior temporal cortex and the CBF value, as well as the compensatory CBF value of the ischemic region (average level of ischemia) and the superior temporal cortex (extreme region of ischemia). Detailed results are presented in Figure 3 and the Supporting Information.

### 3.6 Mediation and Moderation Analysis of Ischemia, Compensation, Gray Matter Abnormality, and MoCA

**FIGURE 4:**
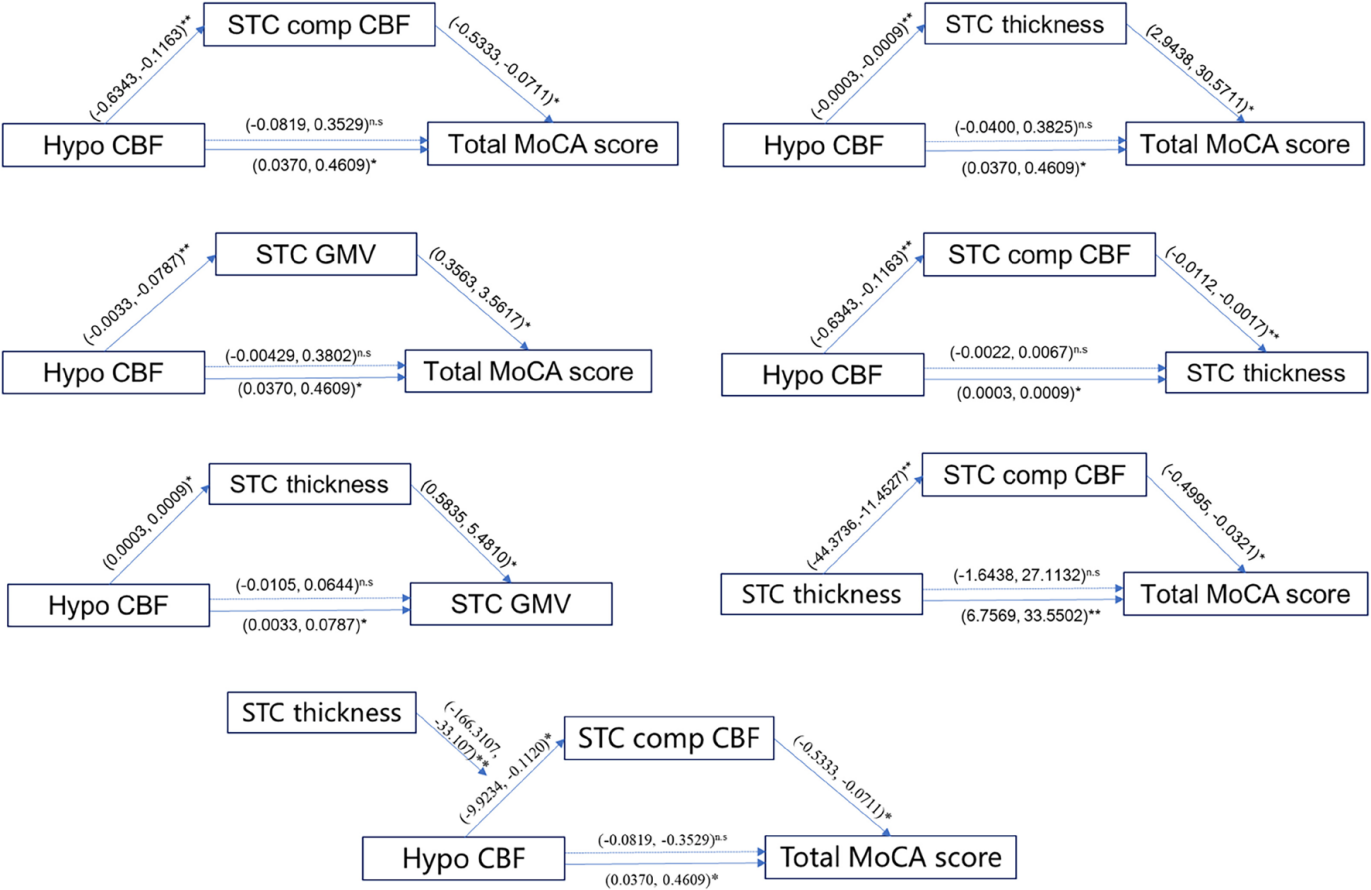
Serial moderation and mediation analyses. Hypo CBF, mean cerebral blood flow in area with significant hypoperfusion; MoCA, Montreal Cognitive Assessment; STC comp CBF, compensatory cerebral blood flow in superior temporal cortex; STC comp CBF, compensatory cerebral blood flow in superior temporal cortex; STC GMV, gray matter volume of superior temporal cortex; STC thickness, cortical thickness of superior temporal gyrus; *, *p* < 0.05; **, *p* < 0.01; n.s., not significant.

PROCESS version 2.16 was used to establish a mediation and moderation model for neuroimaging characteristics and MoCA score. Firstly, superior temporal cortex CBF was shown to have an indirect effect on both its thickness (indirect effect: *b* = 0.0024; direct effect: *b* = 0.0022, *p* = 0.32) and MoCA score (indirect effect: *b* = 0.11; direct effect: *b* = 0.14, *p* = 0.22) via the compensatory CBF of superior temporal cortex. Additionally, superior temporal cortex thickness fully mediated the relationship between ischemic region CBF and MoCA score (indirect effect: *b* = 0.08; direct effect: *b* = 0.17, *p* = 0.11). Superior temporal cortex thickness also had an indirect effect on the MoCA score via superior temporal cortex compensatory CBF (indirect effect: *b* = 7.42; direct effect: *b* = 12.73, *p* = 0.08). Building upon these models, neuroimaging characteristics and MoCA score were integrated into a comprehensive framework. In this framework, superior temporal cortex compensatory CBF mediated the relationship between ischemic region CBF and MoCA score, with superior temporal cortex thickness moderating the effect of ischemic region CBF on superior temporal cortex compensatory CBF (index of moderated mediation: index = −0.69; direct effect: *b* = 0.14, *p* = 0.22, Figure 4).

## 4 Discussion

Understanding of the hypoperfusion impact on collateral circulation development, structural brain changes, and cognitive impairment in MS/TIA patients with ICAS remains limited in clinical practice. Using multimodal MRI and cognitive assessments in this patient cohort, our study yielded the following key findings: (1) cognitive impairment was significantly present and varied in severity, with more pronounced deficits in patients with left hemisphere involvement; (2) extensive regions of hypoperfusion in the involved hemisphere were observed, alongside compensatory hyperperfusion in contralateral and posterior circulation areas; (3) significant alterations were identified in structural morphometry and asymmetry when compared with HC; (4) cognitive impairment associated with ischemia was influenced by structural adaptations and compensatory blood flow, which introduced variability in the relationship between hypoperfusion and cognitive performance.

For patients with chronic cerebral ischemia, particularly those involving intracranial atherosclerosis, focusing on hypoperfusion and its effects is crucial, ideally before a major stroke occurs [23, 24]. Our findings indicate that even among patients presenting with mild ischemic symptoms, extensive hypoperfusion is present in the affected middle cerebral artery territory. This phenomenon highlights the significant impact of large‐artery pathology on cerebral hypoperfusion. We also observed other region‐specific CBF alterations, including abnormally elevated CBF in the posterior circulation territory and, in patients with left hemisphere involvement, in the contralateral hemisphere. The elevated CBF may reflect the activation of a secondary collateral recruitment mechanism, where dormant or newly formed vessels in distant regions are recruited, resulting in a notable increase in CBF in these remote regions [25]. Such brain‐wide perfusion regulation may alleviate hypoperfusion of the involved hemisphere; however, this mechanism may also present an alarming ischemic state [26, 27]. Our correlation and mediation analyses align with previous findings: greater ischemia is correlated with increased collateral recruitment, which may be beneficial but often fails to reverse the ischemic trend, ultimately leaving an avoidable risk of cognitive decline. This indicates that, even if collateral circulation is well‐developed, significant attention should be paid to the underlying ischemic state in these patients.

Hypoperfusion frequently serves as a precursor of cognitive decline across various central nervous system conditions [28]. Unfortunately, most clinical trials focusing on TIA, MS, or ICAS have not focused on cognition outcomes, resulting in a knowledge gap regarding their true functional status. Our study demonstrates that these patients, who are in the close stages of major cerebrovascular events, already show marked cognitive decline across multiple domains, challenging the prevailing clinical view that they experience minimal functional impairment. Importantly, correlation and mediation analysis highlight the robust relationship between ischemic severity and cognitive decline, emphasizing the critical need for cognitive monitoring in the clinical evaluation of cerebral ischemia progression, which enables efficient interventions that prevent further decline and disease advancement.

Beyond the perfusion damage, we observed that these patients exhibited adaptive changes in cortical structures, which are presented only under conditions of severe ischemia [29]. These structural abnormalities, primarily manifesting as significant enlargement of brain volume in severely ischemic regions or their symmetrical counterparts, closely align with the plasticity of involved and contralateral brain regions, as well as an imbalance in interhemispheric inhibitory/excitatory interactions [30, 31]. Consequently, these changes are considered crucial for maintaining and restoring cognitive function following ischemia. Moreover, while cortical abnormalities appear localized, the alteration of gray matter asymmetry suggests it is widespread over the brain, including an AI decrease in patients with left hemisphere involvement and an AI increase in patients with right hemisphere involvement. Referring to the AI formula, the results are correlated with either diminished GMV in the affected cortex or increased GMV in the contralateral cortex [22]. Both of these diverge from the central developing adaptations evolved in humans and probably interrupt the structural advantage that supports cognitive functions [15, 16].

In addition, mediation analysis showed that both GMV and cortical thickness of the ischemic core, that is, the superior temporal cortex, are involved in the development of vascular cognitive impairment. The superior temporal cortex is located near the Sylvian fissure, where the middle cerebral artery branches into the surrounding area within its territory. Distal peripheral blood supply may be insufficient to provide collateral compensation, making ischemia in this area particularly severe. Considering the comprehensive role of the superior temporal cortex in speaking, auditory processing, social perception, and emotional regulation, structural damage to this region is a key pathway by which hypoperfusion induces cognitive impairment. This direct disruption of brain structures, compounded by energy deficits, exacerbates cognitive decline and fosters mechanisms underlying neurodegenerative cognitive disorders [32].

This study confirms that cognitive impairment is prevalent in MS/TIA patients with ICAS and provides further insights into the central mechanisms underlying cognitive impairment. The findings indicate critical perfusion and structural injuries, which are crucial for defining disease characteristics in these patients and advancing secondary prevention strategies. However, this study has certain limitations. First, our sample size was limited, especially for patients with right hemisphere involvement. To ensure cohort consistency, we implemented stringent inclusion criteria, requiring all enrolled patients to undergo digital subtraction angiography to confirm stenosis severity. This process made sample collection more complex and resulted in a small sample size. The small sample size may have reduced statistical power to identify subtle abnormalities or correlations, such as potential structural impairment and cognitive correlation in patients with right hemisphere involvement. Moreover, clinical characteristics differ based on hemisphere involvement, with higher NIHSS scores and higher frequencies of MCA as the offending artery and TIA in patients with right hemisphere involvement. The relatively milder clinical conditions may also explain the insignificant structural and cognitive impairments observed in this cohort. In addition, the data were collected at a single time point, limiting our ability to illustrate the longitudinal trajectory of perfusion deficit, structural abnormalities, and cognitive decline in these patients. Domain‐specific cognitive batteries are needed to uncover more detailed patterns of cognitive impairment. Future work involving larger and more balanced cohorts should focus on longitudinal follow‐ups and therapeutic exploration targeting domain‐specific cognitive and other functional impairments in this cohort.

## 5 Conclusion

Our results demonstrate that ischemia in MS/TIA patients with ICAS is in close connection with marked cerebral structural abnormalities and multidomain cognitive decline. These findings suggest a pressing need for early, targeted interventions aimed at preserving cerebral integrity, mitigating cognitive deterioration, and ultimately reducing the risk of recurrent cerebrovascular events.

## Author Contributions

M.S., G.Y.: data collection, statistical analysis, draft preparation; F.L., S.J.: participant recruitment; L.M., L.Q., X.C., B.G.: data collection; F.P., X.N., W.D.: manuscript revision; H.L., Z.T., P.Y.: draft preparation; J.H., M.Z.: project administration and funding acquisition.

## Funding

This study was funded by grants from the Clinical Research Project Funds for the First Affiliated Hospital of Xi'an Jiaotong University (XJTU1AF‐CRF‐2022‐023), the Fundamental Research Funds for the Central Universities (xzy022023078), Shaanxi Provincial Health Research and Innovation Capacity Enhancement Program (2025TD‐03), and the Key Research and Development Plan of Shaanxi Province (2024SF‐YBXM‐392).

## Ethics Statement

This study was approved by the Institutional Review Board of the First Affiliated Hospital of the Medical College in Xi'an Jiaotong University (XJTU1AF2022LSL‐023), following the requirements of the Helsinki declaration. Informed consent was completely understood and signed by all participants.

## Conflicts of Interest

The authors declare no conflicts of interest.

## Supporting information


**Figure S1:** The comparison results of cortical structure variables between patients and HC and their correlation with the total MoCA score.

## Data Availability

Data are available from the corresponding author upon reasonable request.

## References

[1] D. Deplanque , M. Bastide , and R. Bordet , “Transient Ischemic Attack and Minor Stroke: Definitively Not So Harmless for the Brain and Cognitive Functions,” Stroke 49, no. 2 (2018): 277–278. 29301969 10.1161/STROKEAHA.117.020013

[2] A. Y. Yu and S. B. Coutts , “Stroke: Risk Assessment to Prevent Recurrence After Mild Stroke or TIA,” Nature Reviews Neurology 11, no. 3 (2015): 131–133. 10.1038/nrneurol.2015.16 25686755

[3] M. Sammut , K. Haracz , C. English , et al., “Participants' Perspective of Engaging in a Gym‐Based Health Service Delivered Secondary Stroke Prevention Program After TIA or Mild Stroke,” International Journal of Environmental Research and Public Health 18, no. 21 (2021): 11448. 34769964 10.3390/ijerph182111448 PMC8583419

[4] R. Badacz , T. Przewłocki , I. Karch , et al., “Low Prevalence of Collateral Cerebral Circulation in the Circle of Willis in Patients With Severe Carotid Artery Stenosis and Recent Ischemic Stroke,” Postepy w Kardiologii Interwencyjnej = Advances in Interventional Cardiology 11, no. 4 (2015): 312–317. 26677381 10.5114/pwki.2015.55602 PMC4679799

[5] V. Guglielmi , N. E. LeCouffe , S. M. Zinkstok , et al., “Collateral Circulation and Outcome in Atherosclerotic Versus Cardioembolic Cerebral Large Vessel Occlusion,” Stroke 50, no. 12 (2019): 3360–3368. 31658903 10.1161/STROKEAHA.119.026299 PMC7597992

[6] R. Güney , A. Potreck , U. Neuberger , et al., “Association of Carotid Artery Disease With Collateralization and Infarct Growth in Patients With Acute Middle Cerebral Artery Occlusion,” AJNR. American Journal of Neuroradiology 45, no. 5 (2024): 574–580. 38575322 10.3174/ajnr.A8180 PMC11288550

[7] H. Rowling , D. Italiano , L. Churilov , et al., “Large Vessel Occlusive Stroke With Milder Baseline Severity Shows Better Collaterals and Reduced Harm From Thrombectomy Transfer Delays,” International Journal of Stroke 19, no. 7 (2024): 772–778. 38506406 10.1177/17474930241242954

[8] L. Gallucci , C. Sperber , A. G. Guggisberg , et al., “Post‐Stroke Cognitive Impairment Remains Highly Prevalent and Disabling Despite State‐Of‐The‐Art Stroke Treatment,” International Journal of Stroke 19, no. 8 (2024): 888–897. 38425239 10.1177/17474930241238637

[9] D. Rosenbaum Halevi , A. W. Bursaw , R. R. Karamchandani , et al., “Cognitive Deficits in Acute Mild Ischemic Stroke and TIA and Effects of Rt‐PA,” Annals of Clinical and Translational Neurology 6, no. 3 (2019): 466–474. 30911570 10.1002/acn3.719 PMC6414481

[10] F. J. Wolters , H. I. Zonneveld , A. Hofman , et al., “Cerebral Perfusion and the Risk of Dementia: A Population‐Based Study,” Circulation 136, no. 8 (2017): 719–728. 28588075 10.1161/CIRCULATIONAHA.117.027448

[11] S. Hilal , X. Xu , M. K. Ikram , H. Vrooman , N. Venketasubramanian , and C. Chen , “Intracranial Stenosis in Cognitive Impairment and Dementia,” Journal of Cerebral Blood Flow and Metabolism 37, no. 6 (2017): 2262–2269. 27488908 10.1177/0271678X16663752 PMC5464715

[12] R. S. Marshall , D. S. Liebeskind , J. H. Iii , et al., “Cortical Thinning in High‐Grade Asymptomatic Carotid Stenosis,” Journal of Stroke 25, no. 1 (2023): 92–100. 36592969 10.5853/jos.2022.02285 PMC9911846

[13] X. Leng and T. W. Leung , “Collateral Flow in Intracranial Atherosclerotic Disease,” Translational Stroke Research 14, no. 1 (2023): 38–52. 35672561 10.1007/s12975-022-01042-3

[14] S. Ocklenburg , A. Mundorf , R. Gerrits , E. M. Karlsson , M. Papadatou‐Pastou , and G. Vingerhoets , “Clinical Implications of Brain Asymmetries,” Nature Reviews Neurology 20 (2024): 383–394. 38783057 10.1038/s41582-024-00974-8

[15] L. J. Rogers , P. Zucca , and G. Vallortigara , “Advantages of Having a Lateralized Brain,” Proceedings of the Biological Sciences 271, no. Suppl 6 (2004): S420–S422. 15801592 10.1098/rsbl.2004.0200 PMC1810119

[16] V. Duboc , P. Dufourcq , P. Blader , and M. Roussigné , “Asymmetry of the Brain: Development and Implications,” Annual Review of Genetics 49 (2015): 647–672. 10.1146/annurev-genet-112414-055322 26442849

[17] S. Gao , y. Wang , a. Xu , y. Li , and D. Wang , “Chinese Ischemic Stroke Subclassification,” Frontiers in Neurology 2 (2011): e00006. 10.3389/fneur.2011.00006 PMC3052771 21427797

[18] N. Islam , R. Hashem , M. Gad , et al., “Accuracy of the Montreal Cognitive Assessment Tool for Detecting Mild Cognitive Impairment: A Systematic Review and Meta‐Analysis,” Alzheimer's & Dementia 19, no. 7 (2023): 3235–3243. 10.1002/alz.13040 36934438

[19] U. Fischer , A. Baumgartner , M. Arnold , et al., “What Is a Minor Stroke?,” Stroke 41, no. 4 (2010): 661–666. 20185781 10.1161/STROKEAHA.109.572883

[20] Z. S. Nasreddine , N. A. Phillips , V. Bédirian , et al., “The Montreal Cognitive Assessment, MoCA: A Brief Screening Tool for Mild Cognitive Impairment,” Journal of the American Geriatrics Society 53, no. 4 (2005): 695–699. 15817019 10.1111/j.1532-5415.2005.53221.x

[21] X. Lou , X. Ma , D. S. Liebeskind , et al., “Collateral Perfusion Using Arterial Spin Labeling in Symptomatic Versus Asymptomatic Middle Cerebral Artery Stenosis,” Journal of Cerebral Blood Flow and Metabolism 39, no. 1 (2019): 108–117. 28786338 10.1177/0271678X17725212 PMC6311674

[22] F. Kurth , C. Gaser , and E. Luders , “A 12‐Step User Guide for Analyzing Voxel‐Wise Gray Matter Asymmetries in Statistical Parametric Mapping (SPM),” Nature Protocols 10, no. 2 (2015): 293–304. 25591011 10.1038/nprot.2015.014

[23] D. C. Johnston and M. D. Hill , “The Patient With Transient Cerebral Ischemia: A Golden Opportunity for Stroke Prevention,” Canadian Medical Association Journal = Journal de l'association medicale canadienne 170, no. 7 (2004): 1134–1137. 10.1503/cmaj.1021148 PMC374222 15051699

[24] A. H. Katsanos and R. G. Hart , “New Horizons in Pharmacologic Therapy for Secondary Stroke Prevention,” JAMA Neurology 77, no. 10 (2020): 1308–1317. 32716473 10.1001/jamaneurol.2020.2494

[25] L. Lan , X. Leng , V. Ip , et al., “Sustaining Cerebral Perfusion in Intracranial Atherosclerotic Stenosis: The Roles of Antegrade Residual Flow and Leptomeningeal Collateral Flow,” Journal of Cerebral Blood Flow and Metabolism 40, no. 1 (2020): 126–134. 30351176 10.1177/0271678X18805209 PMC6928549

[26] M. Sebök , C. Niftrik , N. Lohaus , et al., “Leptomeningeal Collateral Activation Indicates Severely Impaired Cerebrovascular Reserve Capacity in Patients With Symptomatic Unilateral Carotid Artery Occlusion,” Journal of Cerebral Blood Flow & Metabolism 41, no. 11 (2021): 3039–3051. 34112002 10.1177/0271678X211024373 PMC8545056

[27] D. A. Lakhani , A. B. Balar , M. Koneru , et al., “The Relative Cerebral Blood Volume (rCBV) < 42% Is Independently Associated With Collateral Status in Anterior Circulation Large Vessel Occlusion,” Journal of Clinical Medicine 13, no. 6 (2024): 1588. 38541813 10.3390/jcm13061588 PMC10971151

[28] Y. Guo , L. Zhou , Y. Li , et al., “Quantitative Transport Mapping of Multi‐Delay Arterial Spin Labeling MRI Detects Early Blood Perfusion Alterations in Alzheimer's Disease,” Alzheimer's Research & Therapy 16, no. 1 (2024): 156. 10.1186/s13195-024-01524-6 PMC11229285 38978146

[29] M. Muller , Y. van der Graaf , A. Algra , J. Hendrikse , W. P. Mali , and M. I. Geerlings , “Carotid Atherosclerosis and Progression of Brain Atrophy: The SMART‐MR Study,” Annals of Neurology 70, no. 2 (2011): 237–244. 21674583 10.1002/ana.22392

[30] S. Xing , E. H. Lacey , L. M. Skipper‐Kallal , et al., “Right Hemisphere Grey Matter Structure and Language Outcomes in Chronic Left Hemisphere Stroke,” Brain: A Journal of Neurology 139, no. 1 (2016): 227–241. 26521078 10.1093/brain/awv323 PMC4990653

[31] Y. Chen , Y. Jiang , X. Kong , et al., “Common and Unique Structural Plasticity After Left and Right Hemisphere Stroke,” Journal of Cerebral Blood Flow and Metabolism 41, no. 12 (2021): 3350–3364. 34415210 10.1177/0271678X211036606 PMC8669287

[32] S. Dong , S. Maniar , M. D. Manole , and D. Sun , “Cerebral Hypoperfusion and Other Shared Brain Pathologies in Ischemic Stroke and Alzheimer's Disease,” Translational Stroke Research 9, no. 3 (2018): 238–250. 28971348 10.1007/s12975-017-0570-2 PMC9732865

