# Supplementary material for: Perfusion and Structural Impairment in Minor Stroke and Transient Ischemic Attack With Intracranial Atherosclerotic Stenosis: Associations With Cognitive Decline
Source: CNS Neurosci Ther. 2025 Dec 9;31(12):e70693. doi: 10.1002/cns.70693 (PMC12690161; doi:10.1002/cns.70693)
Supplement: Supplementary file 1 — Figure S1: The comparison results of cortical structure variables between patients and HC and their correlation with the total MoCA score. [file CNS-31-e70693-s001.docx]

**Perfusion and Structural Impairment in Minor Stroke and Transient Ischemic Attack with intracranial atherosclerotic stenosis: Associations with Cognitive Decline**

# Supporting Information

**Supporting information for the part of methods**

*The parameters for MR sequences*

MR scans were conducted on Discovery MR750 scanners (General Electric, Milwaukee, WI, USA). Earplugs and foam pads were used to diminish the noise and head motion during scanning. The parameters for Sequences of three dimensional T1 brain volume (3D T1 BRAVO) and pseudo-continuous arterial spin labeling (3D pCASL) were set as follows: for 3D T1 BRAVO, repetition time / echo time / inversion time = 8.5ms / 3.2ms / 400ms, flip angle = 12°, slice thickness = 1mm, number of slices = 140, slice gap = 0, matrix = 256 × 256, field of view = 256 × 256 mm^2^; for 3D pCASL, repetition time (TR) = 4642ms for the sequence with post labeling delay (PLD) = 1525ms and TR = 5337ms for the sequence with PLD = 2525ms, echo time = 10.7ms, slice thickness = 4mm, number of slices = 36, slice gap = 4mm, matrix = 128 ×128, field of view = 240 × 240 mm^2^. The combination of ASL sequences with two PLDs enables the observation of the perfusion contribution from both the responsible artery and collateral circulation, detailing the spatial distribution and temporal dynamics of CBF. This approach provides reliable information on the ischemia severity and the extent of compensatory CBF, which benefits a lot in the comprehensive assessment of cerebral perfusion.

Other sequences were also contained in the scanning protocol but did not play a role in the current study.

*Technical procedures for cortical volume measurement*

The technical procedures briefly include removal of non - brain tissue, automated Talairach transformation, segmentation of volumetric structures, normalization, tessellation of the gray matter white matter boundary and automated topology correction. Anatomical ROIs were defined using the fsaverage surface from FreeSurfer. Total intracranial volume (TIV) was extracted out for analysis.

*Statistical analysis*

The distribution of quantitative data was tested by Shapiro - Wilk test. Normally distributed data were described as mean ± SD and compared using independent sample *t* test or analysis of variance. Non - normally distributed data were described as median (interquartile range) and compared using Mann - Whitney *U* test or Kruskal - Wallis H test. Group comparisons were considered statistically significant at *p* < 0.05 for comparisons of total MoCA score and subitems among patients with left / right hemisphere involvement and HC. Post hoc pairwise comparisons were conducted for variables showing significant overall differences among the three groups, and Bonferroni correction was applied to the pairwise results, with a corrected significance level of *p* < 0.0167. For discrete data, frequencies and percentage were presented and Pearson Chi - squared test was used for comparison. SPM12 and Freesurfer were utilized for imaging analysis. AI was compared using one sample *t* test within separate groups. CBF, GMV and AI were compared using independent samples *t* test between left / right involved patients and HC. Multiple liner regression was performed to detect significant correlation between CBF, GMV, AI and total MoCA score in left / right involved patients, respectively. Age, gender, years of education and TIV (not for CBF) were served as covariates and a grey matter mask of whole brain or left hemisphere (for AI) was applied in the comparison and correlation analysis. All results were corrected for family – wise error (FWE) with a cluster - level threshold of p < 0.05. The CBF, compensatory CBF, GMV and thickness of the involved superior temporal cortex (the core of hypoperfusion) were extracted, along with the CBF and the compensatory CBF of the hypoperfusion region. Pearson or Spearman correlation analyses were conducted among these variables and with the total MoCA score according to the distribution of characteristics, in which GMV was standardized as a percentage of TIV. Finally, mediation and moderation analyses were performed to determine the causal relationship between hypoperfusion, collateral perfusion, grey matter abnormality and cognitive decline, during which bootstrap resampling 5000 samples was utilized for internal validation.

**Supporting information for the part of results**

*Single-sample t-test results of asymmetry trends*

A single-sample t-test of HC reveals a rightward AI in the cerebellum posterior lobe, middle occipital cortex, temporal cortex, middle and inferior frontal cortices, as well as anterior and middle cingulate cortices. Leftward AI is found in medial regions, such as thalamus, hippocampus, posterior cingulate cortex, insula, superior temporal cortex, medial frontal cortex, supplementary motor area, and paracentral lobule. In the patient groups, the range of pronounced leftward or rightward AI is relatively narrower compared to the HC, as shown in Fig.4a.

*The correlation between hypoperfusion, compensatory CBF, grey matter atrophy*

Results showed that in the patient group, both the GMV (standardized as the percentage of TIV) and thickness of the affected superior temporal cortex were significantly correlated with its CBF (GMV: P = 0.02, r = 0.348; thickness: *p* < 0.001, r = 0.472), the CBF (GMV: P = 0.009, r = 0.377; thickness: *p* = 0.02, r = 0.336) and the compensatory CBF (GMV: *p* = 0.02, r = -0.338; thickness: *p* < 0.001, r = -0.507) of the hypoperfusion region. The thickness of affected superior temporal cortex was negatively correlated with its compensatory CBF (*p* < 0.001, r = -0.454) and the correlation between GMV of the superior temporal cortex and its compensatory CBF is close to being statistically significant (*p* = 0.051, r = -0.286). The total MoCA score was correlated with both the CBF and the compensatory CBF of the ischemic region (CBF: *p* = 0.04, r = 0.301; compensation: *p* = 0.02, r = -0.337) and the affected superior temporal cortex (CBF: *p* = 0.01, r = 0.374; compensation: *p* = 0.01, r = -0.360). In addition, the total MoCA score was also associated with cortical thickness (*p* = 0.034, *r* = 0.310) and GMV (*p* = 0.011, *r* = 0.368) of the superior temporal cortex. Finally, we verified the correlations between CBF and compensatory CBF in both the superior temporal cortex and the hypoperfusion region. (All *p* < 0.01, see the corresponding r values in Fig.5). All these results were validated by bootstrap with 5000 samples.


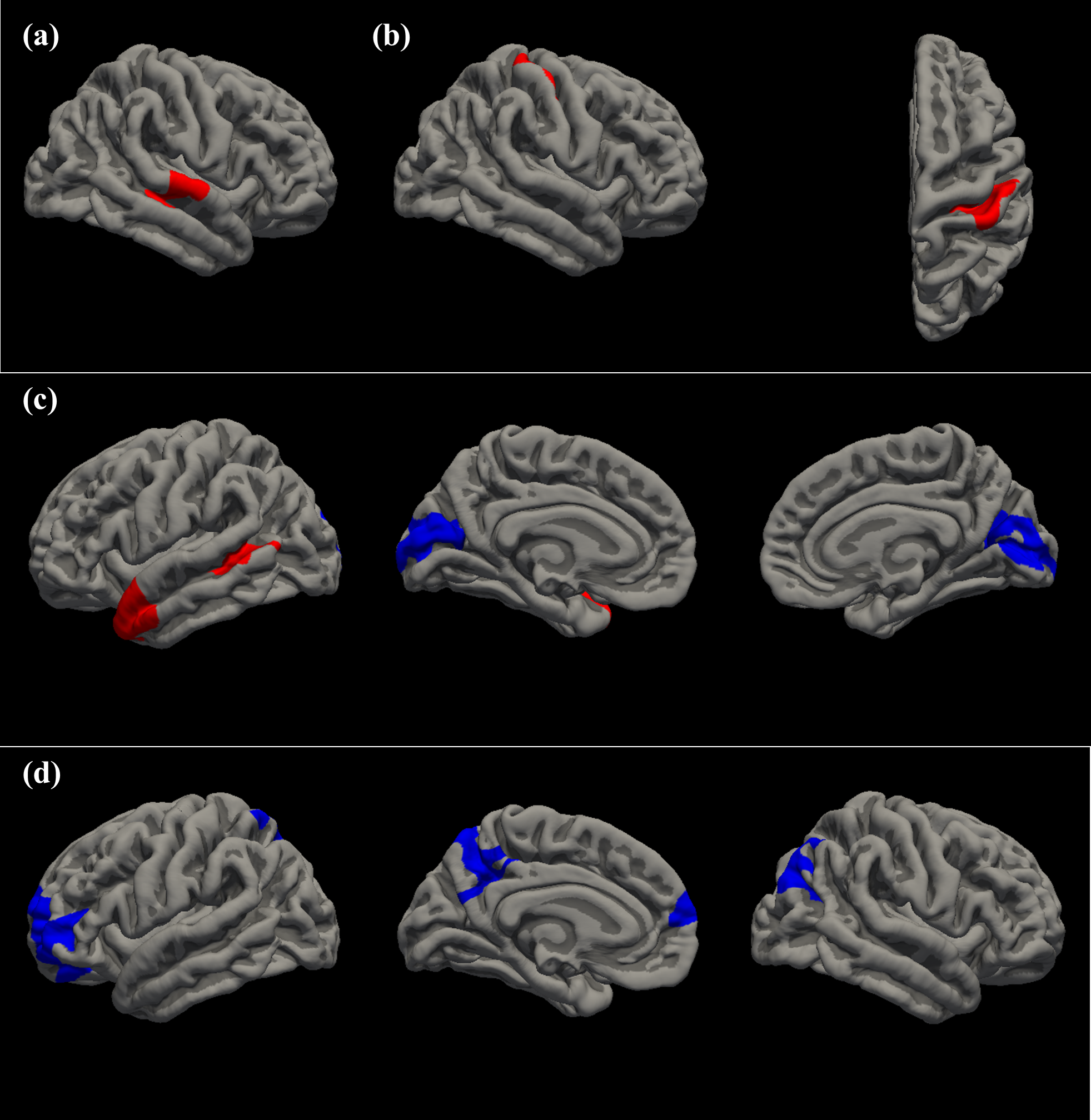


Fig.S1 The comparison results of cortical structure variables between patients and HC and their correlation with the total MoCA score. (a) enlarged clusters in patients with left involvement; (b) enlarged clusters in patients with right involvement; (c) correlation results between total MoCA score and cortical thickness in patients with left involvement; (d) correlation results between total MoCA score and GMV in patients with left involvement. Clusters showing a positive correlation with total MoCA scores are highlighted in red, while those with a negative correlation are highlighted in blue. HC, health controls; MoCA, Montreal Cognitive Assessment; GMV, grey matter volume.

*References*

1. Islam N, Hashem R, Gad M, et al. Accuracy of the Montreal Cognitive Assessment tool for detecting mild cognitive impairment: A systematic review and meta-analysis. *Alzheimer's & dementia : the journal of the Alzheimer's Association* 2023; 19: 3235-3243. 2023/03/20. DOI: 10.1002/alz.13040.
